# Biochemical routes for uptake and conversion of xylose by microorganisms

**DOI:** 10.1186/s13068-020-1662-x

**Published:** 2020-02-01

**Authors:** Zhe Zhao, Mo Xian, Min Liu, Guang Zhao

**Affiliations:** 1 CAS Key Laboratory of Biobased Materials, Qingdao Institute of Bioenergy and Bioprocess Technology, Chinese Academy of Sciences, Qingdao, 266101 China; 2 University of Chinese Academy of Sciences, Beijing, 100049 China

**Keywords:** Xylose, Lignocellulose, Xylose transporter, Xylose catabolic pathways, *Escherichia coli*, *Saccharomyces cerevisiae*, Carbon catabolite repression, Chemicals produced from xylose

## Abstract

Xylose is a major component of lignocellulose and the second most abundant sugar present in nature. Efficient utilization of xylose is required for the development of economically viable processes to produce biofuels and chemicals from biomass. However, there are still some bottlenecks in the bioconversion of xylose, including the fact that some microorganisms cannot assimilate xylose naturally and that the uptake and metabolism of xylose are inhibited by glucose, which is usually present with xylose in lignocellulose hydrolysate. To overcome these issues, numerous efforts have been made to discover, characterize, and engineer the transporters and enzymes involved in xylose utilization to relieve glucose inhibition and to develop recombinant microorganisms to produce fuels and chemicals from xylose. Here we describe a recent advancement focusing on xylose-utilizing pathways, biosynthesis of chemicals from xylose, and engineering strategies used to improve the conversion efficiency of xylose.

## Background

With the increasing concerns regarding the energy crisis and global climate change, it has become of utmost importance to develop clean and sustainable sources for the production of fuels and chemicals. Lignocellulosic biomass is an environment-friendly alternative to fossil energy for fuels and chemicals production and has attracted a great deal of attention, because it is considered the most abundant and non-food-oriented resource. This biomass is a renewable resource by solar energy and carbon dioxide fixation [1, 2]. Lignocellulose has an intricate tridimensional network structure and is composed basically of cellulose (30–50%), hemicellulose (25–30%), and lignin (15–20%). Cellulose is a homopolymer of glucose units interconnected by β 1 → 4 glycosidic bonds, and hemicellulose is a complex branched polysaccharide composed of xylose, arabinose and galactose, among others [3]. Conversion of lignocellulose to biofuels and chemicals requires three main steps: destroying the lignocellulose structure by pretreatment, hydrolyzing hemicellulose/cellulose to fermentable monomeric sugars by enzymatic saccharification, and finally converting monomeric sugars to chemicals or biofuels by microbial fermentation. A major issue in this process is that the majority of fermenting microorganisms cannot metabolize the degradation products of lignocellulose effectively other than glucose [3].

Xylose, derived from hemicellulose, is the second most common sugar in nature and accounts for 18–30% of lignocellulose hydrolysate sugars [4]. Therefore, xylose is considered to be a promising renewable resource for producing biofuels and chemicals. However, to date, there are still some bottlenecks in the process of xylose utilization, resulting in few varieties of products and low conversion efficiency from xylose. First, only a small fraction of microorganisms have the native xylose metabolic pathway [5]. This may greatly limit the selection of engineering strains and the application of xylose. Second, xylose metabolism is severely repressed by glucose in many microorganisms, a process known as carbon catabolite repression (CCR). CCR is a well-known global regulatory phenomenon that allows cells to utilize the most energy-efficient carbon source in a mixture, for example, glucose [6]. This leads to diauxic growth phenomena that affects the metabolism of the secondary sugars derived from lignocellulosic biomass (e.g., xylose) and further limits the conversion efficiency during the fermentation process. Third, transmembrane transport is the first key step for xylose utilization. It has been revealed in some previous studies that the transport rate of xylose limits the downstream pathway flux and that enhancing the transport rate is necessary for improving the cell growth rate and the xylose conversion efficiency [7–9].

Therefore, it is of crucial importance to improve the production of fuels and chemicals from xylose by increasing the xylose uptake efficiency and establishing an effective metabolic pathway. Here, we discuss the aspects regarding the structure and regulation of native xylose transport and catabolism systems in microorganisms as well as the establishment of artificial pathways by introducing heterologous genes. Furthermore, we present strategies to improve the efficiency of xylose uptake and metabolism, such as enzyme engineering and redox balancing. Additionally, some fuels and chemicals produced from xylose by engineered microorganisms and their corresponding pathways are also introduced.

## Xylose uptake

Substrate transport is the first key step in the whole metabolism system during the conversion from lignocellulose to chemicals or fuels. Native sugar transporters, mainly including the ATP-binding cassette (ABC) transporter, the major facilitator superfamily (MFS), and the phosphoenolpyruvate (PEP):carbohydrate phosphotransferase system (PTS), present distinctive substrate specificity and transport mechanism [10]. For example, small soluble molecules are usually transported by the single-polypeptide secondary carriers of MFS with the action of the ion gradient. The MFS is divided into 17 families, and the sugar transport includes families 1, 5, and 7 [11, 12]. Different from the above type, one molecule of ATP per sugar is needed by the ABC transporter, and the phosphate group derived from PEP is transferred to substrate sugar by PTS.

### Native xylose transporters

*Escherichia coli* is one of the most important platform organisms because of its fast growth, clear genetic background, easy genetic manipulation, and low cultivation cost. Moreover, *E. coli* has the native transporters and metabolism pathways of xylose, which confers the ability to use mixed sugars derived from lignocellulosic biomass [13]. Even so, the existence of CCR makes *E. coli* cells preferentially metabolize glucose over xylose [6].

The native xylose transporters of *E. coli* mainly include the MFS protein XylE and the ABC transporter XylFGH. However, the arabinose symporter (AraE) can also play the role of xylose transport in some special situations [14]. The XylE protein is a d-xylose/proton symporter with a relatively low affinity, and the $K_{\text{m}}$ value for xylose is between 63 and 169 μM [13, 15]. Xylose transport by XylE is driven by a proton motive force rather than a direct energy drive or a phosphotransferase, because the addition of xylose to energy-consuming *E. coli* cells can elicit an obvious change of alkaline pH. However, the alkaline pH change fails to appear by uncoupling agents. The uncoupling agents also inhibit the accumulation of [^14^C] xylose in energy-replete cells, whereas arsenates and fluoride do not. The crystal structures of XylE with xylose or glucose (PDB no. 4GBZ) have been obtained, which may provide some valuable insights into the rational design of XylE mutants with improved activity and specificity [16].

The XylFGH protein is a high-affinity d-xylose ABC transporter (the $K_{\text{m}}$ value for xylose is between 0.2 and 4.0 μM) compared with the XylE symporter [15]. Sequence homology analysis suggested that XylF is a periplasmic xylose-binding protein, XylG is an ATP-binding protein, and XylH is a membrane component of the ABC transporter [17, 18]. Among the above systems, XylFGH is the dominant xylose transporter in *E. coli* because the *xylE* knockout mutant showed a much higher growth rate than the *xylG* mutant when xylose was used as the sole carbon source [14].

Clostridia has been widely used for producing acetone, butanol and ethanol (ABE) because of the ability to metabolize a wide range of carbohydrates, such as hexose sugars (glucose, mannose, fructose and galactose), pentose sugars (arabinose and xylose) and disaccharides (lactose and sucrose) [19]. *Clostridium beijerinckii* and *Clostridium acetobutylicum*, the two major species of solventogenic clostridia, have the native xylose transporters and metabolism pathways. However, the xylose utilization efficiency of Clostridia is also poor. The xylose transporters of Clostridia are very similar to those of *E. coli*. The native xylose transporters of *C. beijerinckii* include the sugar-proton symporter (encoded by *xylT*) and d-xylose ABC transporter (encoded by *xylFGH*). However, the genes of ABC transporter are not detected in the chromosome of *C. acetobutylicum*, and only several genes (CAC1339, CAC1345, CAC1530, CAC3422 and CAC3451) encoding sugar-proton symporters are identified [20, 21]. These above symporter genes are distributed in the different parts of the chromosome, and located relatively far apart from each other.

In Archaea, the sugar transporters mainly belong to the class of ABC transporters. Surprisingly, the ABC transporter for d-xylose in Archaea exhibits more similarity to that of bacterium than to any characterized archaeal one. It is revealed that this pentose transporter is the first identified ABC transporter of the carbohydrate uptake transporter-2 (CUT2) family in the domain of Archaea. In addition, the growth defect on d-xylose in the single-gene deletion mutants of ABC transporter further illustrated the importance of this transport system [22]. *Sulfolobus solfataricus* and the closely related *Sulfolobus acidocaldarius* are the best-studied organisms in the phylum of crenarchaeota for sugar transport, and a number of sugar ABC transporters have been identified and examined [23, 24]. Besides those ABC transporters, only the MFS transporter for glucose and PTS system for d-fructose were characterized in *Haloferax volcanii* and *Haloarchaea*, respectively [22].

### Transcriptional regulation of xylose transporter genes

The transcription of the *xylFGH* gene, as well as the *xylAB* gene encoding xylose catabolic enzymes of *E. coli*, is coregulated by a cyclic AMP (cAMP) receptor protein (CRP) and XylR regulator [25]. CRP is a global regulator and exhibits pleiotropic phenotypes by forming a complex with cAMP. The CRP-cAMP complex contributes to CCR and positively regulates the catabolic operons for secondary sugars, such as xylose, galactose, and arabinose, only under glucose-depleted conditions [26, 27]. In order to facilitate xylose uptake and utilization in the presence of glucose, a cAMP-independent mutant CRP* has been discovered and successfully applied in xylitol production from a glucose and xylose mixture [28, 29]. However, the CRP* mutant cannot function in all *E. coli* strains as it did not alleviate glucose repression in *E. coli* B strains FL06 and ATCC 11303 as in *E. coli* K-12 [30]. Moreover, the expression of *xylFGH* and *xylAB* genes is also positively regulated by XylR in response to the d-xylose substrate through direct binding on the corresponding promoters [25]. Unlike XylFGH, the regulatory mechanism of XylE is poorly understood. It is proposed that XylE may also be under the regulation of CRP and XylR [31].

The xylose uptake and metabolism in Clostridia are regulated by a specific repressor XylR and a pleiotropic regulator CcpA [21]. In contrast to the regulatory role in *E. coli*, XylR negatively regulates expression of most genes related with xylose metabolism in *C. beijerinckii*, and deletion of *xylR* results in an obviously improvement for xylose utilization efficiency [32]. However, the *xylR* gene in *C. acetobutylicum* is less certain as compared to that of *C. beijerinckii*, and at least five candidate genes of *xylR* have been raised so far [21]. The expression of xylose uptake and metabolism pathway in *C. acetobutylicum* is directly repressed by CcpA [33]. In addition, the transcription of *xylR* gene is also repressed by CcpA. CcpA also plays an important role in regulating the CCR in gram-positive bacteria [33]. It is demonstrated that *C. acetobutylicum* can co-ferment glucose and xylose in a *ccpA* knockout mutant. So, CcpA may be a potential engineering target for improving the ability of mixed-sugar fermentation of Clostridia.

Although there are several native xylose transporters in different microorganisms, the affinity for xylose is low and actually affects the xylose uptake. Therefore, it is still a long-term and arduous task to improve the xylose transport efficiency by discovering some new native xylose transporters or engineering the known ones.

### Introduction of heterologous xylose transporters in *Saccharomyces cerevisiae*

*Saccharomyces cerevisiae* is also one of most widely used industrial microorganisms. However, wild-type *S. cerevisiae* shows an extremely low cell growth rate with xylose as the sole carbon source due to the lack of efficient and specific xylose transporters [34]. The native hexose transporters in *S. cerevisiae*, which are encoded by *hxt1*–*hxt7* genes, could be considered as candidates for xylose uptake, but these candidates showed much higher affinities to glucose than to xylose [34, 35]. For example, the native high-affinity hexose transporter Hxt7 presents a $K_{\text{m}}$ value for xylose of 130 mM [35]. Fortunately, some efficient xylose transporters were found in other microorganisms. This may be a promising strategy for introducing efficient xylose transporters to the common hosts that cannot transport xylose efficiently.

Filamentous fungi are usually used for screening non-glucose transporters because they can use a multiple of monosaccharides and oligosaccharides and also have the ability to encode a variety of sugar transporters closely related to hexoses and pentoses [36]. For example, *Aspergillus nidulans* is predicted to encode 357 proteins belonging to the MFS, although the number of proteins actually applied to sugar transport is unknown [37]. HxtB, previously considered as a glucose transporter in *A. nidulans*, proved to be a major xylose transporter because the deletion of *hxtB* resulted in a remarkable decline in the xylose uptake efficiency [38], and the monosaccharide transporter XtrD turned out to have a high affinity for xylose [36]. Similarly, *Trichoderma reesei* is predicted to encode 164 proteins belonging to the MFS, which are also candidates for effective xylose transport [39]. Besides, the ortholog of previously identified xylose transporters can be used for further screening. For example, MstA from *Aspergillus niger* is the ortholog of HxtB, and it showed a high affinity for xylose when introduced into *S. cerevisiae* [40].

Until now, a plenty of pentose transporters from filamentous fungi have been successfully expressed in *S. cerevisiae* to improve the xylose uptake and cell growth rate, such as Xut1p, Xut3p, Xut4p, Xut5p, Xut6p, Xut7p, Sut1p, Sut4p, Xyp29p, AraTp, and Rgt2p from *Pichia stipitis*; Gxs1p and Gxf1p from *Candida intermedia*; XylHPp, 2D01474p, and 2C02530p from *Debaryomyces hansenii*; Stp2p, At5g17010p, and At5g59250p from *Arabidopsis thaliana*; Lat1p and Lat2p from *Ambrosiozyma monospora*; and An25p from *Neurospora crassa* [34, 41]. Although so many transporters have been used for the uptake of xylose in *S. cerevisiae*, the results are still unsatisfactory. In the previous analysis, glucose inhibition was still a bottleneck that cannot be ignored in the process of xylose uptake and utilization [42].

## Xylose metabolic pathways

An adequate understanding of natural xylose pathways can provide theoretical guidance for constructing efficient xylose metabolism strains. Currently, many microorganisms can naturally metabolize xylose, including *Escherichia coli*, yeast, filamentous fungi, Caldicellulosiruptor, Clostridia, Proteobacteria, and the domain Archaea [43]. This review listed some typical metabolic pathways of d-xylose.

### Natural metabolism pathways of xylose

**Fig. 1:**
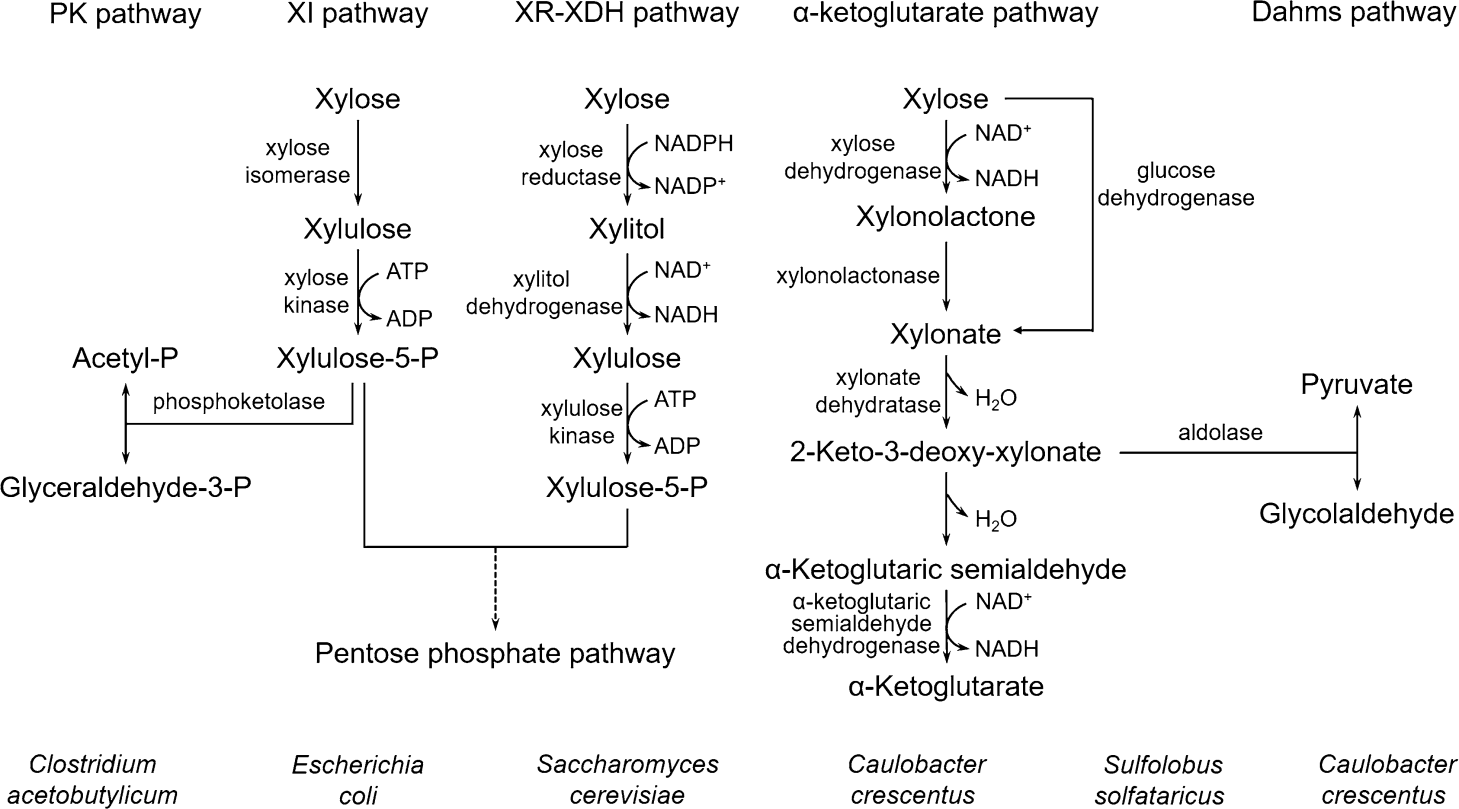
The natural metabolic pathways of xylose in various microorganisms. *PK* phosphoketolase, *XI* xylose isomerase, *XR* xylose reductase, *XDH* xylitol dehydrogenase

In order to make full use of xylose in living environments, microorganisms have evolved distinct xylose utilization pathways over a long period of time (Fig. 1). Xylose is initially converted into xylulose by different enzymes in various microorganisms. In bacteria, isomerization of xylose to xylulose is usually catalyzed by xylose isomerase (XI) directly [44], whereas yeast and mycelial fungi need a two-step pathway employing d-xylose reductase (XR) and xylitol dehydrogenase (XDH) [45]. In this XR-XDH pathway, the first two reactions are usually accompanied with NADPH consumption and NADH production. In both bacteria and fungi, xylulose is further phosphorylated to xylulose-5-phosphate and then metabolized by the pentose phosphate pathway [46]. Furthermore, xylulose-5-phosphate can also be cleaved into acetylphosphate and glyceraldehyde-3-phosphate by phosphoketolase in Clostridia, such as *C. beijerinckii* and *C. acetobutylicum* [21, 47] and *Lactococcus lactis* [48]. In addition to these typical xylose pathways, a new xylose metabolism pathway was discovered in *Caulobacter crescentus*, an oligotrophic freshwater bacterium that employs α-ketoglutarate as a key intermediate [49]. Xylose is converted into xylonolactone and xylonate by NAD^+^-dependent xylose dehydrogenase (Xdh) and xylonolactonase (XylC), respectively. Then, xylonate is dehydrated into 2-keto-3-deoxy-xylonate and α-ketoglutaric semialdehyde, which is oxidized into α-ketoglutarate by α-ketoglutaric semialdehyde dehydrogenase [49]. All enzymes in this xylose metabolism pathway of *C. crescentus* are encoded by the *xyl* operon with xylose induction [50, 51]. Additionally, 2-keto-3-deoxy-xylonate can also be split into glycolaldehyde and pyruvate by the catalysis of an aldolase in the Dahms pathway [52].

The subsequent studies have found that the native xylose metabolism pathways in some Archaea are very similar to the oxidative xylose degradation pathway in *C. crescentus*. For example, the hyperthermophilic archaeon, *Sulfolobus solfataricus* can metabolize glucose to glyceraldehyde and pyruvate via a non-phosphorylative variant of the Entner–Doudoroff pathway with no net ATP yield [53, 54]. In this glucose metabolism pathway of *S. solfataricus*, the first enzyme glucose dehydrogenase and the third enzyme 2-keto-3-deoxygluconate aldolase also show the catalytic activity for pentose. Further investigation reveals that xylose can be directly converted to xylonate by glucose dehydrogenase, and then metabolized to 2-keto-3-deoxy-xylonate by a C5 specific dehydratase. Finally, the 2-keto-3-deoxy-xylonate is split into pyruvate and glycolaldehyde by the C6 and C5 promiscuity aldolase [53]. Furthermore, Johnsen et al. have reported that the xylose metabolism pathway in halophilic archaeon *Haloferax volcanii* shows the highly similarity to that of *C. crescentus*. Xylose is degraded to the key intermediate α-ketoglutarate by sequentially catalytic reaction, involving the enzymes of xylose dehydrogenase (HVO_B0028), xylonolactonase (HVO_B0030), xylonate dehydratase (HVO_B0038A), 2-keto-3-deoxy-xylonate dehydratase (HVO_B0027), and α-ketoglutarate semialdehyde dehydrogenase (HVO_B0039) [55]. The related genes in *H. volcanii* are clustered and transcriptionally regulated by a putative IclR-like regulator HVO_B0040 (designated XacR) [56]. XacR is an activator for pentose catabolism by the validation of mutant and complementation experiments. Besides that, XacR can also negatively regulate its own synthesis [56]. It is the first reported transcriptional regulator for the xylose metabolism in the domain Archaea.

In summary, xylose is converted into different intermediates and eventually metabolized completely for providing the substances and energy for cells. The xylose metabolism in different microorganisms exhibits varying degrees of difficulty but, nonetheless, more than 100 hits for xylose isomerase in the EXPASY database have been listed. It may be the important target for engineering the xylose utilization efficiency.

### Reconstruction of xylose metabolic pathways

Metabolic engineering has been widely used to extend the range or improve the utilization of carbon sources in microorganisms [57]. Nowadays, xylose metabolic pathways have been reconstructed successfully in different microorganisms, such as *S. cerevisiae*, *E. coli*, *Corynebacterium glutamicum*, and *Zymomonas mobilis* [57–60]. As we know, most corynebacteria cannot utilize xylose as a carbon source because of the absence of XI. In order to bring xylose into its substrate utilization range, two recombinant *C. glutamicum* strains were constructed by cloning the *E. coli* XI gene *xylA* either alone or in combination with the *E. coli* xylulose kinase gene *xylB*. Both engineered strains could grow in a mineral medium with xylose as the sole carbon source, and the co-expression strain grew faster on xylose compared with the strain carrying *xylA* alone. Moreover, the resultant strains could simultaneously consume xylose and glucose, demonstrating the absence of diauxic phenomena [59]. These results suggest that the reconstruction of the xylose metabolic pathway in microorganisms that do not utilize xylose naturally could be a useful strategy to derepress glucose inhibition.

Furthermore, the introduction of heterologous xylose metabolic genes can also improve the xylose utilization efficiency of microorganisms that catabolize xylose naturally. Two industrial *S. cerevisiae* strains, NAN-127 and NAN-123, were generated from the parent strain NAN-27 via transforming heterologous xylose-utilizing genes. Both engineered strains showed an enhanced xylose assimilation efficiency. The xylose consumption of strain NAN-127 containing XR and XDH enzymes from *P. stipitis* was two times higher compared with the parent strain, with a 39% improvement in ethanol production. The xylose consumption of strain NAN-123 containing XylB enzyme from *S. cerevisiae* improved by 10% and the ethanol production also improved by 10% compared with that of the parent strain [58].

## Engineering to improve xylose uptake and utilization

Though many microorganisms can naturally metabolize xylose, attempts for improving xylose uptake and utilization were mainly occurred in *E. coli* and *S. cerevisiae*, the most widely used hosts for microbial synthesis. In recent years, there have been rapid developments in metabolic engineering and synthetic biology, allowing the improvement of the uptake and utilization of xylose by engineering the related microorganisms, pathways, and proteins.

### Engineering of xylose transporters

As is known, glucose can competitively inhibit all the previously reported xylose transporters, primarily preventing the simultaneous consumption of xylose and glucose in lignocellulose hydrolysate by microorganisms. Although XylE of *E. coli* is selective for xylose and does not transport glucose, xylose transport is still competitively inhibited by glucose because of the similar high affinities of glucose and xylose to XylE [61]. Much effort has been devoted to the derepression of glucose inhibition, and directed evolution of transporters has proven to be a useful method.

Directed evolution of two transporters, XUT3 from *Scheffersomyces stipitis* and GXS1 from *C. intermedia*, was conducted using yeast growth-based screening, and some mutants with improved properties were obtained. When expressed in *S. cerevisiae*, the mutant transporter improved the cell growth rate on xylose by up to 70% and provided the host strain with a simultaneous sugar utilization capacity in mixed glucose and xylose fermentation [8]. Similarly, the yeast hexose transporters Gal2 and Hxt7 and the *N. crassa* xylose transporter AN25 were engineered via directed evolution to transport xylose efficiently without inhibition by glucose [42, 61].

In directed evolution, a series of mutations at two conserved amino acid residues, threonine 219/213 and asparagine 376/370 in Hxt7 and Gal2, respectively, were identified to abolish glucose inhibition. Among these mutants, Gal2-N376F lost the ability to transport hexoses and showed the highest affinity for xylose [61]. These conserved positions were also found in XylE and other xylose transporters. A model of Gal2 was deduced from the XylE structure, showing that both residues are positioned in the central substrate-binding pocket, although neither directly binds xylose or glucose. N376F mutation impaired hexose transport completely probably because of the larger side chain protruding into the substrate translocation pathway in addition to the changed van der Waals interactions between neighboring amino acids that could alter the structure of the substrate-binding site [61]. Threonine 219/213 is indispensable for glucose transport and may play an important role in binding the C6-hydroxymethyl of glucose [62]. The analysis of conserved amino acids also suggests that N360 in Mgt05196p of *Meyerozyma guilliermondii* is an essential residue for xylose transport [41].

Furthermore, sugar transporter preference and kinetics can be rewired through the programming of a conserved sequence motif G–G/F-XXX-G (X represents a variable but usually nonpolar amino acid residue) in the first transmembrane span. This motif is highly conserved among functional sugar transporters. After saturation mutagenesis and subsequent rational mutagenesis, several hexose transporters acquire a high affinity for xylose, which can enable them to grow on xylose but not on glucose when expressed in a *S. cerevisiae* strain lacking endogenous monosaccharide transporters. The GGFIMG motif was found to have the highest specificity and efficiency for xylose probably because the larger side chains physically restrict the size of the pore, making it easier for smaller xylose molecules to bind and traverse compared with larger hexoses [9]. This study helps increase the understanding of the structure–function relationships of sugar transporters, providing novel insights into alleviating the effect of CCR, and it establishes a platform for engineering a specific and efficient xylose transporter.

All these results greatly promoted the construction of engineered strains that can simultaneously consume glucose and xylose. For instance, Gal2-N376F was introduced into *Kluyveromyces marxianus*, and the resultant strain produced ethanol and xylitol with a yield of 0.42 g ethanol/g glucose and 0.99 g xylitol/g xylose, respectively, in the co-fermentation of glucose and xylose [63].

### Recycling of cofactors between XR and XDH

In fungi, xylose catabolism begins with its conversion into xylulose by XR and XDH (Fig. 1). However, there is a difference in the cofactor specificity of XR (preferential with NADPH) and XDH (strictly with NAD^+^), resulting in a limited supply of NAD^+^ for XDH, accumulation of xylitol, and reduced production of fuels or chemicals [64]. Altering the coenzyme specificity and realizing the effective recycling of cofactors between XR and XDH have been widely performed for the improvement of the xylose fermentation efficiency.

XR encoded by the *C. parapsilosis XYL1* gene is the first reported XR to prefer NADH [65]; it carries an arginine instead of a lysine in the IPKS motif conserved in NADPH-dependent XRs [66]. In order to mimic the *C. parapsilosis* XR, a K270R mutant of *P. stipitis* XR was made and expressed in a recombinant *S. cerevisiae* strain. In continuous fermentation, the strain carrying the *P. stipitis* XR K270 mutant gave a xylitol yield of 0.05 g/g xylose, whereas the strain with wild-type *P. stipitis* XR gave a xylitol yield of 0.24 g/g xylose [67]. The coenzyme specificity of *P. stipitis* XR has also been altered successfully by a semirational approach. Based on a homolog model deduced from the *C. tenuis* XR structure, six residues in *P. stipitis* XR were predicted to interact with the adenine ribose of NAD(P)H and become altered by saturation mutagenesis. The best mutant, carrying four mutations—K270S, S271G, N272P, and R276F—showed 13-fold improved preference for NADH compared with NADPH. In contrast, the cofactor specificity of XDH can be completely reversed to NADP^+^. Four amino acid residues in *P. stipitis* XDH—Asp207, Ile208, Phe209, and Asn211—were selected for site-directed mutagenesis to examine their roles in the discrimination between NAD^+^ and NADP^+^. Finally, the quadruple mutant (D207A/I208R/F209S/N211R) presented a $k_{\text{cat}} /K_{\text{m}}$ value with NADP^+^ 4500-fold higher compared with the wild-type enzyme, reaching a value comparable to $k_{\text{cat}} /K_{\text{m}}$ with NAD^+^ of the wild-type protein. As the thermostability of the NADP^+^-dependent XDH mutant decreased, an additional zinc-binding site composed of three cysteine residues was introduced into the quadruple mutant to improve the protein thermostability, which further increased the catalytic activity with NADP^+^ fourfold [68]. The quadruple mutant was introduced into recombinant *S. cerevisiae* along with *P. stipitis* XR, and 86% decreased unfavorable xylitol accumulation and 41% increased ethanol production was found compared to the control strain carrying the wild-type XDH [69].

### Evolutionary engineering for improving xylose metabolism

In order to obtain the xylose-utilizing strains, a series of metabolic engineering modifications have been performed. However, it was found that the xylose-utilizing pathway is obviously affected by the different backgrounds of strains and growth conditions [70], complicating the integration of knowledge from various researches for rational design and construction. Evolutionary engineering can globally modulate metabolic networks without extensive regulatory or genetic information about the desirable phenotypes, appropriate for improving the utilization of unfavorable carbon sources.

*Escherichia coli* MG1655 was engineered to produce d-lactate from xylose by the deletion of the *pflB*, *adhE*, *frdA*, and *xylFGH* genes [71]. Knockout of *pflB*, *adhE* and *frdA*, involved in production of by-product formate, ethanol and succinate, may improve the xylose conversion efficiency. The *xylFGH* genes encoding ATP-dependent xylose transporter was deleted to increase ATP availability. In order to further improve cell growth and lactate productivity, this strain was applied to adaptive evolution on xylose. The evolved strain showed a 50% increase in the growth rate and lactate productivity and a twofold increase in the xylose consumption rate. Whole-genome sequencing revealed a point mutation in the GatC protein, which resulted in the replacement of serine by leucine at position 184. This modified GatC protein can be used as a new xylose transporter that improves the growth and consumption rate of xylose in that evolved strain [71].

A recombinant *S. cerevisiae* strain KF7M-16, carrying the *P. stipitis* XR-XDH pathway, was also subjected to adaptive evolution. The evolved strain YNBX26-21 showed significantly improved cell growth and ethanol production rate. Comparative transcriptome analysis revealed novel responses to xylose, including the biosynthesis of vitamins B1 and B6 and sulfur amino acid and decreased expression of Fe(II) transport-related genes and several glucose-repressible genes, which probably contributed to the improved xylose utilization [72]. All these results from adaptive evolution provided new insights into the construction of a superior xylose-utilizing strain through inverse metabolic engineering.

## Biosynthesis of chemicals from xylose

In recent years, the bioconversion of xylose into value-added chemicals has attracted considerable attention. Many wild microbial strains or engineered strains have been identified or constructed to utilize xylose for producing a variety of compounds, including ethanol, 1,4-butanediol (BDO), 1,2,4-butanetriol (BT), ethylene glycol (EG), glycolate, xylitol [29, 63], and succinate [73, 74] (Table 1).

**Table 1:** Metabolism of xylose in different recombinant microorganisms for high-value chemicals and biofuels production

Strain	Substrate	Chemicals	Titer (g/L)	Yield (g/g)-xylose	Productivity (g/L/h)	References
*Z. mobilis*	Xylose	Ethanol	43.1	0.45	0.94	[77]
*S. cerevisiae*	Xylose	Ethanol	16.4	0.41	0.77	[89]
*S. cerevisiae*	Xylose	Ethanol	10.8	0.25	0.39	[90]
*S. cerevisiae*	Xylose	Ethanol	10.4	0.26	0.24	[70]
*E. coli*	Xylose	BT	5.1	0.255	0.106	[81]
*E. coli*	Xylose	BT	3.92	0.20	0.05	[78]
*E. coli*	Xylonate	BT	1.6	0.25		[80]
*E. coli*	Xylose	BT	0.88	0.13	0.012	[91]
*E. coli*	Xylose	BDO	0.209	0.01	0.004	[82]
*E. coli*	Xylose	EG	108.2	0.36	2.25	[88]
*E. coli*	Xylose	EG	72	0.40	1.38	[87]
*E. coli*	Xylose	EG	40	0.35	0.56	[83]
*E. coli*	xylose	EG	20	0.38	0.37	[86]
*E. coli*	Xylose	Glycolate	44	0.44	0.92	[83]
*E. coli*	Xylose	Glycolate	43.6	0.46	0.91	[84]
*K. lactis*	Xylose Ethanol	Glycolate	14.8	0.28	0.09	[92]
*E. coli*	Xylose	Glycolate	4.57	0.46	–	[93]
*E. coli*	Xylose	Glycolate	4.3	0.46	0.089	[94]

### Ethanol

Ethanol is a promising biochemical that is widely used as a gasoline additive and biofuel. In recent years, *S. cerevisiae* have been widely used for ethanol production because they are highly tolerant to ethanol [75]. Besides the strategies of xylose-utilizing pathway reconstruction, redox balancing, and adaptive evolution described above, an efficient, simple, and programmable approach for the rapid tuning of gene expression in a heterologous pathway, named “customized optimization of metabolic pathways by combinatorial transcriptional engineering (COMPACTER)”, was used to improve ethanol productivity from xylose in recombinant *S. cerevisiae* carrying the xylose reductase from *Candida shehatae* (csXR), the xylitol dehydrogenase from *Candida tropicalis* (ctXDH) and the xylulose kinase from *Pichia pastoris* (ppXKS) [70]. Specifically, each gene in the xylose-utilizing pathway was assembled with promoter mutants of varying strengths to construct a library of mutant pathways, which was subjected to high-throughput screening. After a single round of COMPACTER, the optimized mutant produced ethanol 1.5 times faster and consumed xylose 70% faster compared with the reference strain carrying the same xylose metabolic pathway under the control of wild-type promoters.

Besides *S. cerevisiae* systems, other microorganisms were also developed to produce ethanol from xylose, such as *E. coli*, *Z. mobilis*, and *P. stipitis*. Among these, *Z. mobilis* is one of the best ethanol producers found in nature owing to their high theoretical ethanol yield from sugars and high ethanol productivity rate [76]. However, *Z. mobilis* do not have the native xylose metabolism pathway. In order to expand their substrate range to xylose, two operons encoding *E. coli* XylAB and PPP enzymes were cloned and transformed into *Z. mobilis*, conferring the co-fermentation of glucose and xylose to ethanol [60]. To further improve xylose fermentation, adaptive evolution involving 30 transfers in a medium containing a high concentration of xylose was conducted, and a strain A3 finally obtained the significant improvement of xylose metabolism. This strain was able to grow on 100 g/L xylose and rapidly fermented xylose to ethanol within 2 days. The final titer of ethanol was 43.1 g/L with a yield of 0.45 g/g xylose and ethanol productivity of 0.94 g/L/h [77]. These results revealed that metabolic engineering and adaptive evolution could be used synergistically for strain improvement.

### BT and BDO

BT is an important fine chemical and has versatile applications in many fields. For example, it can be used for making polyurethane foam, which has compression-bending properties similar to natural rubber. As a potential building block, BT is also used for synthesizing various pharmaceuticals and is used as a direct precursor for BT trinitrate, a great energetic plasticizer [78]. BDO is a valuable commodity chemical, with an annual market of an excess of 2.5 million tons worldwide. As a chemical intermediate, BDO goes into an extraordinarily wide spectrum of products, including automotive parts, electronics, and apparel [79].

**Fig. 2:**
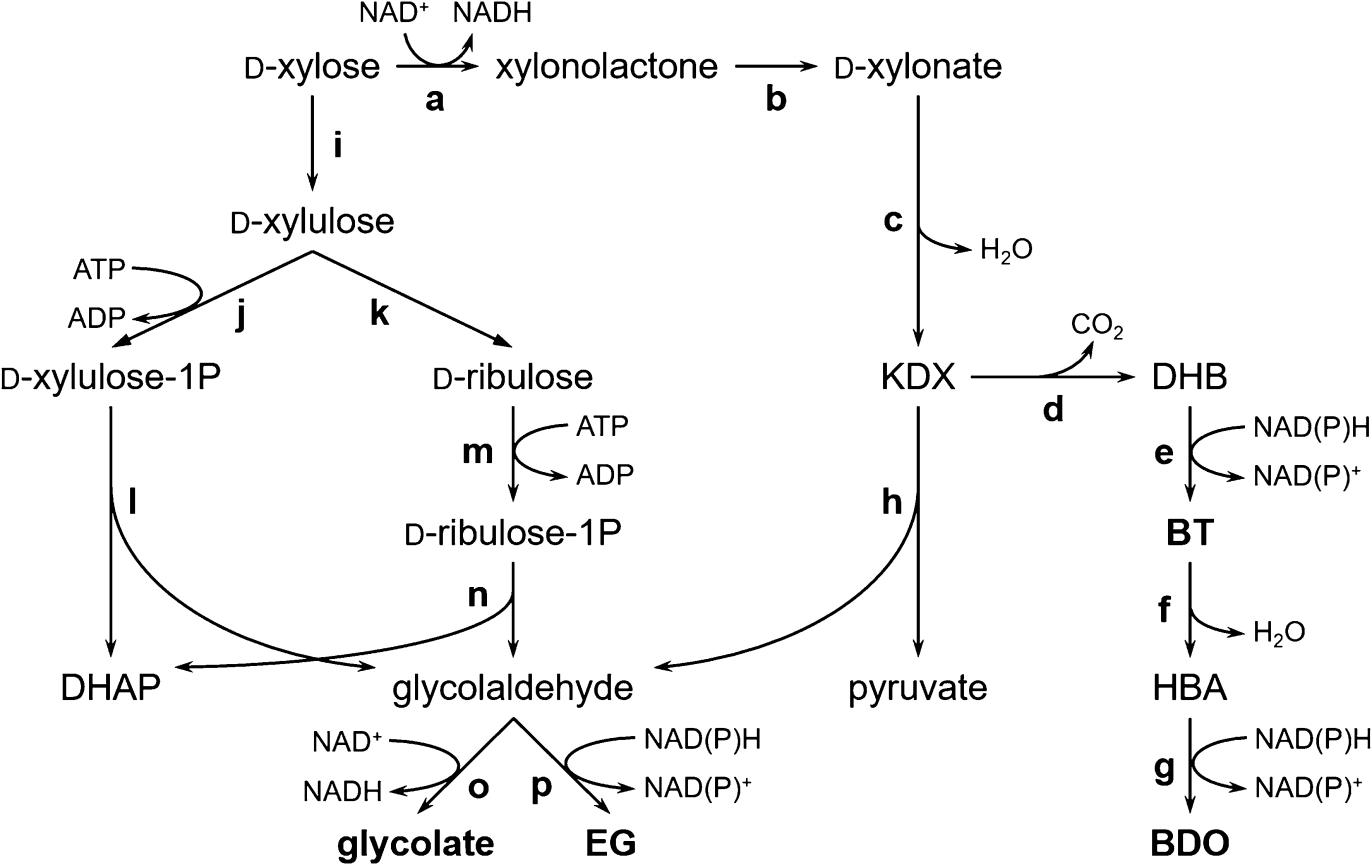
Biosynthetic pathways of chemicals from xylose. DHAP, dihydroxyacetone phosphate; EG, ethylene glycol; KDX, 2-keto-3-deoxy-d-xylonate; DHB, d-3,4-dihydroxybutanal; BT, d-1,2,4-butanetriol; HBA, 4-hydroxybutyraldehyde; BDO, 1,4-butanediol. a d-xylose dehydrogenase, XlyB from *C. crescentus*; b xylonolactonase, XylC from *C. Crescentus*; c d-xylonate dehydratase, YjhG/YagF from *E. coli*, XylD from *C. crescentus*; d decarboxylase, KivD from *Lactococcus lactis*, MdlC from *Pseudomonas putida*; e alcohol dehydrogenase, YqhD/AdhP from *E. coli*, ADH2 from *S. cerevisiae*; f diol dehydratase, engineered PpdA-C-B fusion from *Klebsiella oxytoca*; g alcohol dehydrogenase, YqhD from *E. coli*; h 2-keto-3-deoxy-d-pentanoate aldolase, YjhH/YagE from *E. coli*; i d-xylose isomerase, XylA from *E. coli*; j d-xylulose-1-kinase, Khk-C from human; k d-tagatose 3-epimerase, DTE from *Pseudomonas cichorii*; l d-xylulose-1-phosphate aldolase, Aldo-B from human; m l-fuculokinase, FucK from *E. coli*; n l-fuculose-1-phosphate aldolase, FucA from *E. coli*; o aldehyde dehydrogenase, AldA from *E. coli*; p alcohol dehydrogenase, FucO/YqhD from *E. coli*

The first report on the biosynthesis of BT employed two microbial strains. Xylose is initially converted to xylonate by *Pseudomonas fragi*, and the *E. coli* construct is responsible for the conversion of xylonate into BT, which carries a 2-keto acid decarboxylase gene *mdlC* from *Pseudomonas putida* (Fig. 2; [80]. However, the production and purification of xylonate is a tedious process that increases the whole production cost of BT. Thus, a recombinant *E. coli* strain was constructed to directly convert xylose into BT [78]. The Xdh XylB and XylC from *Caulobacter crescentus* and 2-keto acid decarboxylase MdlC from *P. putida* were co-expressed with the native xylonate dehydratase YjhG and aldehyde reductase AdhP in *E. coli* BL21 star (DE3) (Fig. 2). Meanwhile, the endogenous XI XylA and xylulose kinase XylB were deleted to block the native xylose metabolism pathway. The final constructed strain produced 3.92 g/L BT from 20 g/L xylose, corresponding to a yield of 0.20 g/g xylose. A similar BT-producing strain was constructed using aldehyde reductase YqhD instead of AdhP. In combination with the knockout of 2-keto acid reductase YiaE to eliminate the competing branch pathway, this strain produced 5.1 g/L BT with a yield of 0.26 g/g xylose [81].

Based on the BT-producing route, a de novo biosynthetic pathway of BDO was established (Fig. 2; [82]. The *Klebsiella oxytoca* diol dehydratase, which dehydrates its native substrate 1,2-propanediol efficiently, was rationally engineered to promote catalysis toward a nonnative triol, BT. The mutant enzyme showed dehydratase activity toward BT by nearly fivefold in comparison to the wild-type protein. By introducing the complete pathway into the *E. coli* strain, the biosynthesis of BDO at 209 mg/L from xylose was achieved.

### EG and glycolate

EG is a commodity chemical that can be used in multiple everyday applications. There are two most prominent uses of EG: automotive antifreeze and one of the precursors for poly(ethylene terephthalate). Moreover, EG can be converted into acetaldehyde with the help of diol dehydratase and then transformed into ethanol, acetate, or acetyl-CoA [83]. Glycolate is an organic acid containing both carboxyl and alcohol groups. It is an ideal choice for a wide range of applications, such as a precursor for biopolymer synthesis, a rinsing agent in the tanning and dyeing industry, and a skincare product in the cosmetics industry. Additionally, the glycolate polymer is a new type of packaging material with excellent performance because of its gas-barrier and mechanical properties [84]. With a growing demand, the global market of glycolate is expected to reach USD 277.8 million in 2020 (http://www.grandviewresearch.com/press-release/-glycolic-acid-market).

The biosynthetic pathways for EG and glycolate were successfully reconstructed in *E. coli*. They are both derived from the intermediate glycolaldehyde, which can be produced from xylose via three different routes (Fig. 2). In the Dahms pathway, the metabolite 2-keto-3-deoxy-xylonate can be split into glycolaldehyde and pyruvate by *E. coli* endogenous pentanoate aldolase YjhH/YagE [84, 85]. In contrast, xylose is initially isomerized to xylulose, which is further transformed into xylulose-1-phosphate or ribulose-1-phosphate in different recombinant strains [83, 86]. Then, pentose-1-phosphate is split into glycolaldehyde and dihydroxyacetone phosphate by Aldo-B from human or FucA from *E. coli* (Fig. 2). The conversion of xylose into each mole of glycolaldehyde via the Dahms pathway generates one mole of NADH, whereas one mole of ATP is consumed in both routes with xylulose-1-phosphate and ribulose-1-phosphate as intermediates.

In order to improve the productivity of EG and glycolate, strategies including redox balancing [87], elimination of competitive branch pathways [86], and overcoming acetate overflow [84, 87] were performed. Up till now, the highest titers of EG and glycolate produced from xylose are 108 g/L with a yield of 0.36 g/g xylose [88] and 44 g/L with a yield of 0.44 g/g xylose [83], respectively.

In summary, all these results fully confirm the great potential of xylose as a carbon source for the biosynthesis of high-value chemicals and biofuel. It is useful to extensively use xylose to fully explore lignocellulose resources and provide a new direction for microbial fermentation.

## Conclusion

As the second most abundant sugar in lignocellulose, xylose is a promising renewable resource for producing biofuels and chemicals, and much effort has been expended in xylose bioconversion. A number of xylose transporters from *E. coli* and filamentous fungi were discovered and characterized, and five xylose assimilation pathways were identified in various microorganisms. In microorganisms that cannot metabolize xylose naturally, such as *S. cerevisiae*, artificial pathways were reconstructed by the introduction of heterologous xylose transporter and catabolic genes. Value-added chemicals, including ethanol, BT, BDO, EG, and glycolate, were synthesized from xylose by recombinant microorganisms, and the productions were significantly improved through metabolic engineering. Despite the achievements that have been made, there are still some bottlenecks in xylose bioconversion. First, glucose represses the activity of xylose transporters as well as the transcription of xylose-related genes, hampering the co-utilization of glucose and xylose in lignocellulose hydrolysate. It is necessary to focus on further developments in order to explore new xylose transporters without glucose inhibition and establish cAMP-CRP-independent transcriptional regulation for genes involved in xylose utilization. Second, the conversion efficiency of xylose to target chemicals is low, resulting in a high production cost. We believe that the yield should increase significantly in the future, benefiting from the development of new technologies in enzyme discovery and optimization, fine-tuning of the expression level of multiple genes in a complex pathway, and evolutionary engineering, among other methods.

## Data Availability

Not applicable.

## Abbreviations

*S. cerevisiae*: *Saccharomyces cerevisiae*
*E. coli*: *Escherichia coli*
*A. nidulans*: *Aspergillus nidulans*
*Z. mobilis*: *Zymomonas mobilis*
CCR: Carbon catabolite repression
ABC: ATP-binding cassette
MFS: The major facilitator superfamily
PTS: Carbohydrate Phosphotransferase System
XI: Xylose isomerase
XR: Xylose reductase
XDH: Xylitol dehydrogenase
PPP: Pentose phosphate pathway
PK: Phosphoketolase
BDO: 1,4-Butanediol
BT: 1,2,4-Butanetriol
EG: Ethylene glycol
